# Effect of Formulation Factors and Oxygen Levels on the Stability of Aqueous Injectable Solution Containing Pemetrexed

**DOI:** 10.3390/pharmaceutics12010046

**Published:** 2020-01-06

**Authors:** Dong Han Won, Heejun Park, Eun-Sol Ha, Yong Min Kim, Hyung Don Hwang, Sun Woo Jang, Min-Soo Kim

**Affiliations:** 1 Dong-A ST Co. Ltd., Giheung-gu, Yongin, Gyeonggi 446-905, Korea; w3921@donga.co.kr (D.H.W.); kdragonm@donga.co.kr (Y.M.K.); hd0823@donga.co.kr (H.D.H.); chb@donga.co.kr (S.W.J.); 2 College of Pharmacy, Pusan National University, 63 Busandaehak-ro, Geumjeong-gu, Busan 46241, Korea; pharmacy4336@pusan.ac.kr (H.P.); edel@pusan.ac.kr (E.-S.H.)

**Keywords:** pemetrexed, stability, aqueous injectable solution, oxidation, control strategy

## Abstract

The aim of this study was to investigate the effects of various parameters at each control strategy in drug product degradation on the stability of pemetrexed in injectable aqueous solution. A forced degradation study confirmed that oxidation is the main mechanism responsible for the degradation of pemetrexed in aqueous solutions. As control strategies, the antioxidant levels, drug concentration, pH of the control formulation, dissolved oxygen (DO) levels in the control process, and headspace oxygen levels in the control packaging were varied, and their effects on the stability of pemetrexed were evaluated. Sodium sulfite was found to be particularly effective in preventing the color change, and *N*-acetylcysteine (NAC) had a significant effect in preventing chemical degradation. The sulfite and NAC were found to stabilize pemetrexed in the aqueous solution by acting as sacrificial reductants. A pH below 6 caused significant degradation. The stability of pemetrexed in the solution increased as the concentration of the drug increased from 12.5 to 50 mg/mL. In addition, the DO levels in the solution were controlled by nitrogen purging, and the oxygen levels in headspace were controlled by nitrogen headspace, which also had significant positive effects in improving the stability of the pemetrexed solution; thus, it was confirmed that molecular oxygen is involved in the rate-limiting oxidation step. Based on these results obtained by observing the effects of various control strategies, the optimal formulation of an injectable solution of pemetrexed is suggested as follows: sodium sulfite at 0.06 mg/mL, as an antioxidant for prevention of color change; NAC at 1.63 mg/mL, as an antioxidant for prevention of chemical degradation; pH range 7–8; DO levels below 1 ppm; and headspace oxygen levels below 1%. In conclusion, it can be suggested that this study, which includes well-designed control strategies, can lead to a better understanding of the complex degradation mechanism of pemetrexed; thus, it can lead to the development of an injectable solution formulation of pemetrexed, with improved stability.

## 1. Introduction

Pemetrexed disodium heptahydrate, *N*-(4-(2-(2-Amino-3,4-dihydro-4-oxo-7H-pyrrolo(2,3-d)pyrimdin-5-yl)ethyl)benzoyl)glutamic acid·2Na·7H_2_O, is a synthetic folate analog metabolic inhibitor which can disrupt folate-dependent metabolic processes that are required for cell replication, and it has been used in the treatment of various cancers [1]. Alimta^®^ (Eli Lilly and Company, Indianapolis, IN, USA) containing pemetrexed disodium heptahydrate as an active ingredient was registered by the US Food and Drug Administration (FDA) for the treatment of mesothelioma in 2007 [2,3]. It is formulated as a lyophilized cake in a single-dose vial that is reconstituted with 0.9% sodium chloride injection, to make a solution of pemetrexed at a concentration of 25 mg/mL prior to administration. The appropriate volume of reconstituted pemetrexed solution should be diluted further to 100 mL with 0.9% sodium chloride injection and administered as an IV infusion over 10 min. All procedures for the reconstitution and infusion of solution should be performed immediately because there are no antimicrobial preservatives present in the formulation [4]. In addition, the chemical and physical stability of the reconstituted pemetrexed solution has been demonstrated to last only for up to 24 h after the reconstitution of the original vial, when stored at controlled room temperature [4,5]. Owing to the disadvantages of such a lyophilized injection, there has been a requirement for the development of a ready to use (RTU) pemetrexed liquid injection; however, the relatively rapid formation of degradants has been the main challenging factor so far in developing it as a commercially available final drug product [6,7,8,9].

The mechanism of degradation has been reported previously [7]. Under acidic conditions, decarboxylation of glutamic acid is observed, and des-glutamate and glutamic acid are generated. Under alkaline conditions, degradation proceeds by hydrolysis of the side-chain amide, followed by deamination. In the presence of oxygen, three oxidative degradants, α-hydroxy lactams, keto-pemetrexed, and oxidative dimers are formed by the oxidation pathway. Although several studies have attempted to improve the instability of pemetrexed in aqueous solution state, none of them have provided useful information that can be utilized in actual product development.

In general, following parameters can affect the stability of injectable drug solutions: temperature, exposure to light, pH of the solution, presence of additives, concentration of API, presence of oxygen, and duration of storage [10,11,12,13,14,15,16,17,18,19]. In order to stabilize the injectable drug solution, control strategies have been attempted after thorough understanding of the critical quality attributions (CQA), critical material attributes (CMA) and critical process parameters (CPP) in relation to the formulation and manufacturing process, and considering the quality of the intermediate and final products [20]. Thus, the control strategies can be categorized into (i) control of excipients, (ii) control of formulation, (iii) control of process, and (iv) control of packaging [21,22].

The basis for the content criteria of related substances is to establish a validated quantification method and select acceptable criteria range for individual and total impurity in accordance with the International Council for Harmonisation of Technical Requirements for Pharmaceuticals for Human Use (ICH) guidelines [23,24,25]. Extensive stability screening studies that are conducted based on well-designed experiments are required to establish the content criteria of related substances, and to develop a stable formulation that can meet the criteria.

To the best of our knowledge, before this study, there was no systematic study conducted on the stabilization of pemetrexed solution by using multiple approaches in various ways according to the recommendation by the ICH guidelines. Owing to limited data available from previous reports, well-designed control strategies, following a greater understanding of the degradation of pemetrexed, are necessary because they would contribute to the improvement of the stability of the injectable solution formulations of pemetrexed, which in turn may increase the utilization and safety of this drug.

Thus, the aim of this study was to evaluate the stability of pemetrexed in aqueous solution under various control strategies, to acquire beneficial information for the minimization of degradation without color change. First, a forced degradation study was performed to determine the degradation mechanism, including the main pathway responsible for the decomposition of pemetrexed in solution state. Of the four control strategies mentioned above, three were attempted, except for the category (i) control of excipients, which was not attempted because an auto-oxidation susceptible excipient was not used for the preparation of pemetrexed injectable solution in this study. The effects of pH, drug concentration, and addition of antioxidants were evaluated as the formulation controls. In addition, the dissolved oxygen (DO) levels in the solution controlled by nitrogen purging and oxygen level in headspace controlled by nitrogen headspace were evaluated as controls of process and packaging, respectively. The decrease in drug content and the increase in degradation products were monitored by quantitative methods using HPLC, and the change in color due to decomposition was measured by a colorimetric method. In addition, the amounts of antioxidants consumed by oxidation were measured by their quantification methods, and a correlation with the degradation pattern of pemetrexed was investigated to evaluate the potential of antioxidants as sacrificial reductants.

## 2. Materials and Methods

### 2.1. Materials

Pemetrexed disodium hemipentahydrate was purchased from Nippon Kayaku (Tokyo, Japan). d-mannitol was obtained from Roquette (Corby, UK). Hydrochloric acid (HCl), sodium hydroxide (NaOH), hydrogen peroxide, potassium dihydrogen phosphate, ortho-phosphoric acid (PA), sodium carbonate, sodium bicarbonate, sodium formaldehyde bisulfite, sodium sulfate, and sodium sulfite were purchased from Merck (Darmstadt, Germany). The pemetrexed-related substances, α-hydroxy lactams, keto-pemetrexed, oxidative dimers, and ring-opened keto-amide were obtained from TRC (Toronto, ON, Canada). *N*-acetylcysteine (NAC, Kyowa Hakko Bio Co., Ltd., Tokyo, Japan), l-cysteine·HCl, sodium sulfide, and sodium metabisulfite were purchased from Sigma Aldrich (Saint Louis, MO, USA). Hydroxypropyl-β-cyclodextrin (Kleptose^®^ HPB, Roquette-Freres, Lestrem CEDEX, France) and d-α-tocopheryl polyethylene glycol succinate (Vitamin E TPGS, Eastman Chemical Co., Kingsport, TN, USA) were also utilized. Acetonitrile and acetone were obtained from Fisher Scientific (Pittsburgh, PA, USA). All other chemicals were of reagent grade and used without further purification.

### 2.2. Analytical Method of Pemetrexed Impurities

Reverse-phase high-performance liquid chromatography (RP-HPLC) was performed to analyze the contents of pemetrexed and its related substances. RP-HPLC was conducted as previously described, but with slight modifications [7]. HPLC analysis was performed by using a Dionex Ultimate 3000 RS HPLC system (Dionex Corporation, Sunnyvale, CA, USA) equipped with an Ultimate 3000 RS diode array detector and pump, as well as an RP C8 column (Zobrax RX-C8, 5 µm, 4.6 × 250 mm, Agilent Technologies, Palo Alto, CA, USA). The auto-sampler and column temperature were maintained at 4 and 30 °C, respectively. The injection volume was 10 µL, and the flow rate was maintained at 1.0 mL/min. Two mobile phases were prepared for the gradient elution. Mobile phase A was composed of 1.36 g/L potassium dihydrogen phosphate at pH 2.5, adjusted by 85% ortho-phosphoric acid. Mobile phase B was composed of acetonitrile. The elution program for the binary gradient system is shown in Table 1. UV detection was performed at 277 nm. The calibration curve of pemetrexed showed a good linearity over the concentration range of 78–121 µg/mL and was used in this study with a correlation coefficient (R^2^) of 0.999. The content (%) of impurities was calculated by the following equation based on the United States Pharmacopeia (USP) monograph of pemetrexed for injection [26,27]:

(1) $$\mathrm{Content}\;\mathrm{of}\;\mathrm{impurity}\;\left(\%\right)=\;\frac{A_{U}}{A_{T}}\;\times \;\frac{1}{\mathrm{RRF}}\;\times \;100$$

where A_U_ is the peak area of each impurity from the sample solution, A_T_ is the total peak area from the sample solution, and RRF is the relative response factor for each impurity. The total impurity content was obtained based on the sum of the peak areas corresponding to all impurities, and RRF_i_ = 1.

### 2.3. Colorimetric Determination

The color change by degradation was evaluated by color measurement, using a colorimeter (Konica-Minolta CM-3500d spectrocolorimeter, Konica Minolta, Osaka, Japan). The b*, one of the chromaticity coordinates in color space defined by International Commission on Illumination (CIE), was measured by using the D_65_ illuminant and 10° standard observer, using 2 mL plastic cuvettes. Based on the chromaticity diagram of color space, positive value of b* indicates the change to yellowish colors and negative value of b* indicates the change to bluish colors.

### 2.4. Forced Degradation Test

Forced degradation tests using various decomposition pathways were carried out as follows: Mannitol was dissolved in water for injection (WFI) at a concentration of 100 mg/mL. The drug stock solution was prepared by dissolving pemetrexed disodium hemipentahydrate in mannitol solution at a concentration of 100 mg/mL. Each test solution was prepared by following the method described below for each degradation condition and being filtered immediately through 0.2 µm Nalgene filter units (Nalgene, Rochester, NY, USA) for sterilization, followed by filling 20 mL of test solution in a glass vial and sealing with a cap. The vials containing test solution were stored at specific conditions, considering various decomposition mechanisms, and the samples were withdrawn at predetermined time intervals, and then they were diluted suitably and analyzed by HPLC. Each experiment was performed in quadruplicates.

#### 2.4.1. Thermal Degradation

The pH of the stock solution was adjusted to 7.0 ± 0.1 by the addition of 0.1 N of NaOH, and the stock solution was then diluted to a final drug concentration of 25 mg/mL, using WFI. The prepared test sample vials were secondary packed, using a box to block the light, and then stored at 60 °C for 4 weeks.

#### 2.4.2. Acidic Hydrolysis

The test solution was prepared by dilution of the stock solution to a final drug concentration of 25 mg/mL, using 0.01 N of HCl. The prepared test sample vials were secondary packed, using a box to block the light, and then stored at 25 °C for a day.

#### 2.4.3. Basic Hydrolysis

The test solution was prepared by dilution of the stock solution to a final drug concentration of 25 mg/mL, using 1 N of NaOH. The prepared test sample vials were secondary packed, using a box to block the light, and then stored at 25 °C for a day.

#### 2.4.4. Oxidation

A 6% solution of hydrogen peroxide, a strong oxidizing agent, in WFI, was added to the two times diluted stock solution, at a volume/volume ratio of 1:1. The final concentration of hydrogen peroxide and pemetrexed was 3% and 25 mg/mL, respectively. The prepared test sample vials were secondary packed, using a box to block the light, and then stored at 25 °C for a day.

#### 2.4.5. Photostability

The test solution was prepared by dilution of the stock solution to a final drug concentration of 25 mg/mL, using WFI, and then the vials containing the test solution samples were stored in a photostability chamber equipped with one ultraviolet lamp with 22 W UV power, at 25 °C, for 1 day, in accordance with the Guidance for Industry Q1B Photostability Testing of New Drug Substances and Products by ICH [28].

### 2.5. Effect of Control Strategy Parameters on Pemetrexed Stability

According to the control strategies recommended in the ICH guidelines [21], the effects of (i) antioxidants and pH in formulation, (ii) solution oxygen levels in process parameters, and (iii) headspace oxygen levels in packaging parameters, on the stability of pemetrexed, were evaluated. The experimental methods for each category are described below in detail. After a certain storage period under each specific condition, samples were withdrawn, and the collected samples were analyzed by colorimeter. For quantification, the collected samples were filtered through 0.2 µm polyvinylidene fluoride (PVDF) syringe filter (Whatman GmbH, Dassel, Germany), and all filtered solution samples were diluted suitably and then analyzed by HPLC. All experiments were performed in quadruplicates.

#### 2.5.1. Antioxidant as a Parameter for Control of Formulation

The stability of pemetrexed solution with or without antioxidants was evaluated at 60 °C for 3 weeks. The used antioxidants and their concentrations are presented in Table 2.

The experimental methods and ordering are as follows: (i) mannitol was dissolved in WFI, followed by nitrogen purging, using high-tensile 316 stainless-steel tubing (HIP, Erie, PA, USA) and a gas-flow regulator (Chiyoda Seiki, Seoul, Korea) to decrease the oxygen levels to 1.0 ± 0.2 ppm in the solution. The DO levels in the solution were measured by using a portable Orion Star A326 DO meter (Thermo Scientific, Chelmsford, MA, USA); (ii) the antioxidants were dissolved in mannitol solution, and the pH was preadjusted to 5.0 ± 0.1, by addition of 0.5 N of HCl or 0.5 N of NaOH; (iii) pemetrexed was dissolved in the above solution and mixed; (iv) the pH was adjusted to 7.0 ± 0.1 by the addition of 0.5 N of HCl or 0.5 N of NaOH, (v) after pH equilibration, and the final test solutions were prepared by dilution to desired concentrations of the drug and antioxidants, using WFI, as presented in Table 2. Each test solution was filtered through 0.2 µm Nalgene filter units (Nalgene, Rochester, NY, USA), for sterilization. Then, 20 mL of the filtered test solution was filled in the glass vials, followed by nitrogen headspace substitution by using high-tensile 316 stainless-steel tubing (HIP, Erie, PA, USA) and a gas-flow regulator (Chiyoda Seiki, Seoul, Korea), to reduce the headspace oxygen levels in the vial to 1.0 ± 0.2%. After the nitrogen headspace, the vial was sealed with a cap. The headspace oxygen content was determined by frequency-modulated spectroscopy (FMS-760, Lighthouse Instruments, Charlottesville, VA, USA). The sealed vials containing the test solution were stored at 60 °C for 3 weeks.

In order to evaluate the effects of various antioxidant types, sulfur, including oxidation-reducing agents, phenolic vitamin derivative, and inclusion complex forming agent were selected (Table 2). Sodium sulfide was selected as sulfides; sodium sulfite and sodium metabisulfite were selected as sulfides; and NAC and l-cysteine-HCl were selected as sulfur-containing amino acids. In addition, vitamin E TPGS and HP-β-CD were selected as phenolic vitamin derivative and inclusion-complex-forming agent, respectively. In order to evaluate the effect of antioxidant concentration on the stability of the pemetrexed solution, the final concentrations of sodium sulfite and NAC in the sample solution were varied from 0.03 to 0.12 mg/mL and from 1.00 to 1.63 mg/mL. These concentration ranges of various antioxidants were selected based on the clinically prescribed concentration for injection use under FDA approval [30,31,32]. The vials containing the test solutions were stored at 60 °C for 3 weeks.

To evaluate the potential of sodium sulfite as a sacrificial reductant, a test solution with a sodium sulfite concentration of 0.12 mg/mL (76.2 mg/mL as a sulfite) was prepared, and then the vial containing the test solution was stored at 25 °C for 22 months, for long-term stability testing. The amounts of sulfite consumed by oxidation and the sulfate generated by oxidation were measured by high-performance ion chromatography (HPIC) [33]. The test solution for NAC was prepared at a concentration of 1.63 mg/mL, and then the vial containing the test solution was stored at 25 °C for 22 months, for long-term stability testing. The NAC amount consumed by oxidation was determined by the RP-HPLC method. The initial antioxidant concentration before storage was compared to that measured after long-term stability testing. Then, the results were compared with the degradation pattern of pemetrexed and were further analyzed to determine the correlation between them.

#### 2.5.2. Drug Concentration as a Parameter for Control of Formulation

The effect of drug concentration on the stability of pemetrexed was evaluated by using two samples containing 0.06 mg/mL of sodium sulfite and 1.63 mg/mL of NAC as antioxidants. At pH 7, final drug concentrations of 50, 25, and 12.5 mg/mL were prepared by diluting with WFI (Table 2). The prepared test sample vials were stored at 40 °C for 4 weeks, and at 60 °C for 4 weeks.

#### 2.5.3. pH as a Parameter for Control of Formulation

The effect of pH on pemetrexed stability was evaluated to determine the optimal pH value for the stability of pemetrexed in aqueous solution. Considering the limited solubility of pemetrexed in acidic aqueous solvents, the evaluation was performed over the pH range of 6–8.5. The pH of the test solutions was adjusted to 6, 6.5, 7, 7.5, 8, and 8.5 in the pH adjustment step iv. The final concentrations of drug, sodium sulfite, and NAC were 50, 0.06, and 1.63 mg/mL, respectively (Table 2). The prepared sample vials were stored at 40 °C for 4 weeks and at 60 °C for 3 weeks.

#### 2.5.4. Dissolved Oxygen (DO) Level in Solution as a Parameter for Control of Process

In order to evaluate the effect of oxygen levels in solution on the stability of pemetrexed, two samples with different DO levels, 1 ± 0.2 ppm and 7 ± 0.2 ppm, were prepared by nitrogen purge according to the step (i) in the aforementioned method for evaluating antioxidant effect. The minimum DO that can be controlled accurately by the self-made nitrogen headspace substitution apparatus used in this study was 1 ± 0.2 ppm, so 1 ± 0.2 ppm was set as the lowest DO level. These samples contained 50 mg/mL of pemetrexed, 0.06 mg/mL of sodium sulfite and 1.63 mg/mL of NAC. The prepared sample vials were stored at 60 °C for 4 weeks.

#### 2.5.5. Nitrogen Headspace as a Parameter for Control of Packaging

The effect of oxygen levels in the headspace on the stability of pemetrexed was evaluated. The concentrations of the drug, sodium sulfite, and NAC in the test solution were 50, 0.06, and 1.63 mg/mL, respectively (Table 2). The oxygen level in the headspace for the test samples varied from 0.2% to 2.5% by nitrogen headspace at step vi. The prepared sample vials were stored at 40 °C for 4 weeks.

### 2.6. Analysis Method of Sulfite and Sulfate

To evaluate the potential of sodium sulfite as the sacrificial reductant, the amount of sulfite consumed by oxidation and the sulfate generated by oxidation was measured and compared before and after the evaluation of Section 2.5.1. In addition, the results were further compared with the degradation pattern of pemetrexed and analyzed to find a correlation between them. HPIC was used to simultaneously determine sulfite (SO_3_^2−^) and its oxidized form, sulfate (SO_4_^2−^) [33]. The HPIC method was slightly modified from the previously described method. HPIC was performed with suppressed conductivity detection, using a Dionex Ultimate 3000 RS HPLC system (Dionex Corporation, Sunnyvale, CA, USA) equipped with an anion micro-membrane suppressor (AMMS, Dionex, Sunnyvale, CA, USA), a conductivity detector (CDM, Sunnyvale, CA, USA), and an anion-separation column (Shodex IC SI-90 4E, 4.0 × 250 mm, Showa Denko KK, Tokyo, Japan). The auto-sampler and column temperature were maintained at 4 and 30 °C, respectively. The injection volume was 10 µL, and the flow rate was maintained at 1.0 mL/min. Isocratic mobile phase was composed of 3 mM of NaHCO_3_, 1.5 mM of Na_2_CO_3_, and 5% acetone in deionized water (DW). Sodium formaldehyde bisulfite and sodium sulfate were used as the standards for sulfite and sulfate, respectively, and the conversion amounts for sulfite and sulfate were calculated by multiplying molecular weight ratio after purity correction. The calibration curve of sulfite and sulfate concentration vs. area showed a good linearity over the concentration range of 18.1–362.0 µg/mL, with a correlation coefficient (R^2^) of 1.00.

### 2.7. Analysis Method of NAC

RP-HPLC was performed to analyze the consumed amount of NAC by oxidation. The results were further compared with the degradation pattern of pemetrexed and analyzed to determine a correlation between them. RP-HPLC analysis was carried out on a Dionex Ultimate 3000 RS HPLC system (Dionex Corporation, Sunnyvale, CA, USA) equipped with an Ultimate 3000 RS diode array detector and RP C18 column (µBondapak C18, 125Å, 10 µm, 3.9 × 300 mm, Waters Ass., Milford, MA, USA). The auto-sampler and column temperature were maintained at 4 and 30 °C, respectively. The injection volume was 5 µL, and the flow rate was maintained at 1.5 mL/min. Two mobile phases were prepared for gradient elution (Table 3). Mobile phase A was composed of 50 mM of KH_2_PO_4_, with pH 3.0. Mobile phase B was composed of acetonitrile. The elution program for the binary gradient system is presented in Table 3. UV detection was performed at 214 nm [34].

### 2.8. Statistical Analysis

To evaluate the statistical significance of the differences between groups, Student’s *t*-test or one-way analysis of variance (ANOVA), followed by Tukey’s HSD test and SNK test, was carried out by using SPSS 25.0 software (SPSS, Chicago, IL, USA).

## 3. Results and Discussions

### 3.1. Forced Degradation Test

The forced degradation behavior of pemetrexed under various stress conditions, such as thermal stress degradation, acidic and basic hydrolyses, and peroxide and photo-induced oxidations, was evaluated in the aqueous state. Identification of the impurities in the forced degradation samples was performed according to the relative retention time (RRT) between each impurity and pemetrexed [7,8]. The RRT of the representative degradation products obtained in this study and reported previously is shown in Table 4. It was reported that the α-hydroxy lactams, keto-pemetrexed, also known as lactam isomers, and oxidative dimers can be generated by oxidization reaction of pemetrexed with singlet oxygen or free radical, which is typically observed in the oxidized drug products. In particular, the oxidative dimers were the most frequently produced oxidative decomposition products in all the conditions. However, the opened-ring keto-amide and keto-formamide can be generated by heating [7].

**Figure 1:**
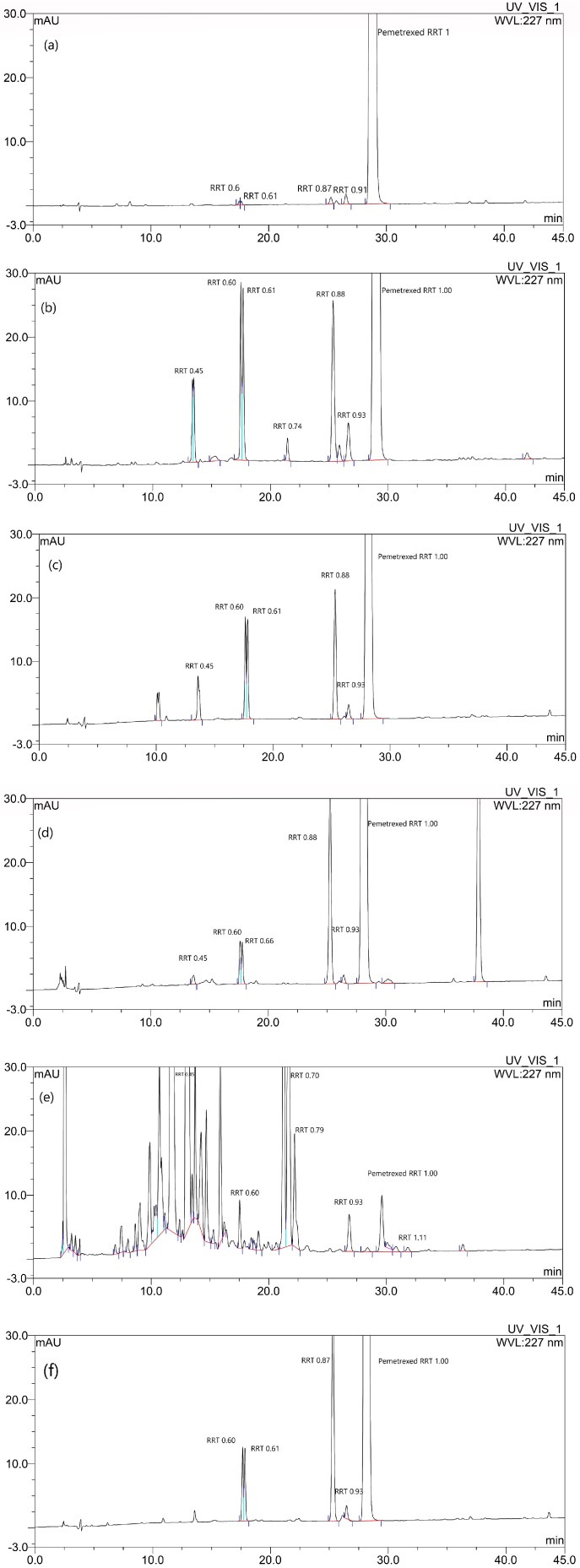
HPLC chromatograms of pemetrexed solution samples: (**a**) initial standard; (**b**) thermal stressed; (**c**) acidic hydrolysis; (**d**) basic hydrolysis; (**e**) oxidation by hydrogen peroxide; and (**f**) photo-oxidation.

Most of the degradation products formed under thermal stressed conditions are supposed to result from the oxidative mechanism, but a relatively large increase in the opened-ring keto-amide, which can be generated from heating, was observed as compared to the other samples (Figure 1b) [8]. Predicting the degree to which heat could accelerate oxidation is difficult because of the inverse relationship between the amount of dissolved oxygen and the temperature [29]. Thus, at higher temperatures, there is less dissolved oxygen but a faster intrinsic reaction rate and potentially more initiation [21]. Therefore, it is assumed that the generation of oxidative decomposition products of pemetrexed due to thermal stress occurs very clearly in this study. The hydrolytic pathway can occur in acidic conditions (Figure 1c); the des-glutamate and glutamic acid are formed from the acid-catalyzed hydrolysis of the amide linkage of pemetrexed. This hydrolytic degradation product could be proceeded to further degradation by oxidation [7,8]. As NaOH was added, oxidation became the dominant degradation mechanism, with the oxidative dimers being the major degradation products detected under these conditions (Figure 1d). As shown in Figure 1e, it is apparent that the change in HPLC chromatogram, characterized by the appearance of peak resulting from degradation-related substances, was the most pronounced among the results for the sample stress by the peroxide induced oxidation. The pemetrexed solution exposed to simulated sunlight in photostability chamber also gave rise to a number of degradation products, keto-pemetrexed, and oxidative dimers, resulting from oxidation (Figure 1f) [7]. From these results, most of the degradation products formed under various forced degradation conditions are proposed to result via an oxidative mechanism. Therefore, it was found that the focus should be on the prevention of the instability induced by oxidation in order to improve the aqueous solution stability of pemetrexed.

### 3.2. Control of Formulation

#### 3.2.1. Effect of Antioxidants

**Figure 2:**
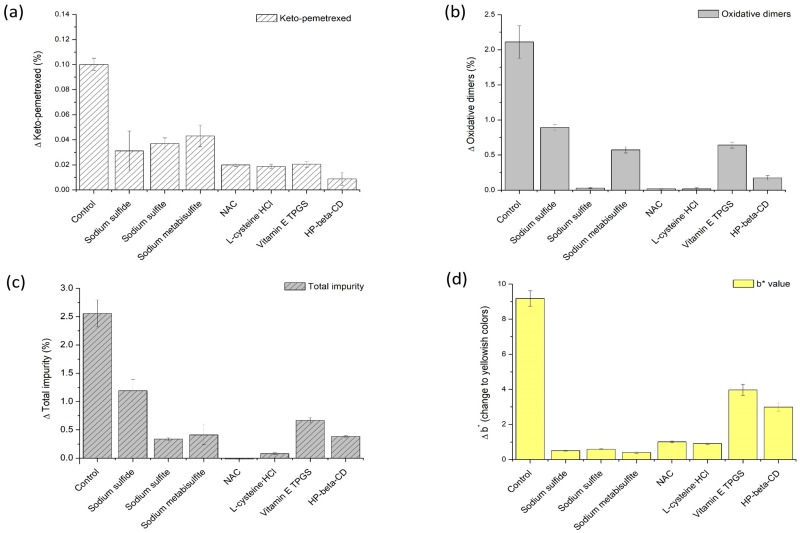
Effect of antioxidants on pemetrexed degradation induced by stress condition at 60 °C in aqueous solution: (**a**) increase in keto-metotrexed content; (**b**) increase in oxidative dimer content; (**c**) increase in total impurity content; and (**d**) increase in b* value as an indicator of color change to yellowish.

The effect of antioxidants on the pemetrexed degradation induced by stress condition at 60 °C in aqueous solution is shown in Figure 2. All used antioxidants inhibited not only pemetrexed degradation but also color change to yellowish, as represented by the b* value. Sulfur-containing amino acids such as NAC and l-cysteine·HCl were more effective at preventing chemical degradation than the other antioxidants, and NAC had the greatest stabilization effect. However, sulfur-containing sulfides and sulfites were more effective at inhibiting the color change than amino acids, with sodium sulfite being the most effective at preventing chemical degradation among them. Consequently, sodium sulfite and NAC were selected as the final antioxidant combination based on both color change and degradation product results, from among the candidates including sulfites, amino acids, and other antioxidants. Vitamin E TPGS and HP-β-CD were relatively less effective for stabilization of pemetrexed solution, and they were excluded from the final formulation.

**Figure 3:**
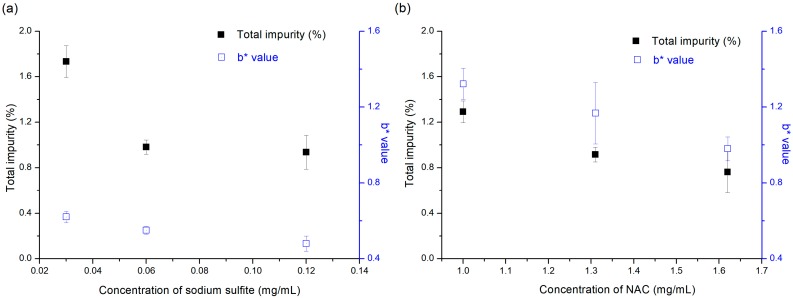
Effect of antioxidant concentration on the total impurity (■) and color change (□) generated by stress condition at 60 °C for 3 weeks in aqueous solution: (**a**) sodium sulfite and (**b**) NAC.

The effect of the concentrations of selected antioxidants, sodium sulfite and NAC, on pemetrexed degradation is shown in Figure 3. The b* values of pemetrexed solution at various sodium sulfite concentrations were much less than that of NAC, which again confirms that sodium sulfite is more effective at inhibition of color change than NAC, despite being added in relatively small amount. When pemetrexed solutions containing sodium sulfite at various concentrations as an antioxidant were stored at 60 °C for 3 weeks, the total impurity decreased in the following order: 0.03 mg/mL > 0.06 mg/mL = 0.12 mg/mL (ranked by the SNK test, *p* < 0.05). There was no significant increase observed in the stabilization effect by increasing the sodium sulfite concentration above 0.06 mg/mL; hence, a concentration of 0.06 mg/mL sodium sulfite was determined to be optimal. The total impurity (%) and b* values, after stability testing at 60 °C for 3 weeks, of pemetrexed solutions containing NAC at various concentrations as ranked by the SNK test were as follows (*p* < 0.05): 1.00 mg/mL > 1.32 mg/mL > 1.63 mg/mL, which presents a stabilization effect that is linearly proportional to its concentration. The optimal concentration of NAC was determined to be 1.63 mg/mL, considering the upper limit of the clinically available range as an inactive ingredient for injectable drug product.

**Figure 4:**
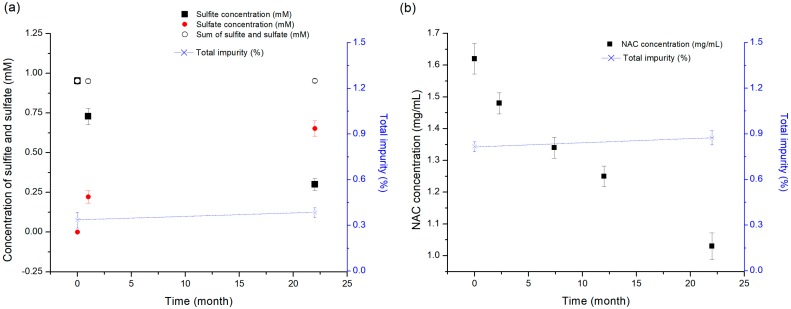
Ability of sodium sulfite (0.12 mg/mL) and NAC (1.63 mg/mL) as sacrificial reductants (25 °C for 22 months): (**a**) changes in total impurity (╳) and in concentrations of sulfite (■), oxidized sulfate (●) and their sum (○), with storage period; (**b**) changes in total impurity (╳) and in concentrations of NAC (■) with storage period.

The amounts of sodium sulfite and NAC consumed by oxidation were measured by their quantification methods, and their correlations with the degradation patterns of pemetrexed were investigated to evaluate the potential of the antioxidants as sacrificial reductants. For sodium sulfite, sulfate generated by oxidation was also measured to compare with the amount of consumed sulfite [33]. Sacrificial reductants are compounds that are oxidized more readily than the drug. They effectively scavenge oxygen, while they are themselves consumed; hence, they inhibit the oxidation of the drug [17,21]. As shown in Figure 4a, the concentration of sulfite decreased while the concentration of sulfate increased over the storage period. Furthermore, the sum of the sulfite and sulfate remained the same within the evaluation period, suggesting that sulfite was oxidized to sulfate in solution over time. Based on these results, the content of pemetrexed and impurity did not increase significantly because of oxidation of the sulfite instead of pemetrexed. NAC also continued to decrease in proportion to storage time, while the content of pemetrexed was maintained. Consequently, it was confirmed that sodium sulfite and NAC can act as sacrificial reductants to prevent the degradation of pemetrexed in aqueous solution.

#### 3.2.2. Effect of Drug Concentration

**Figure 5:**
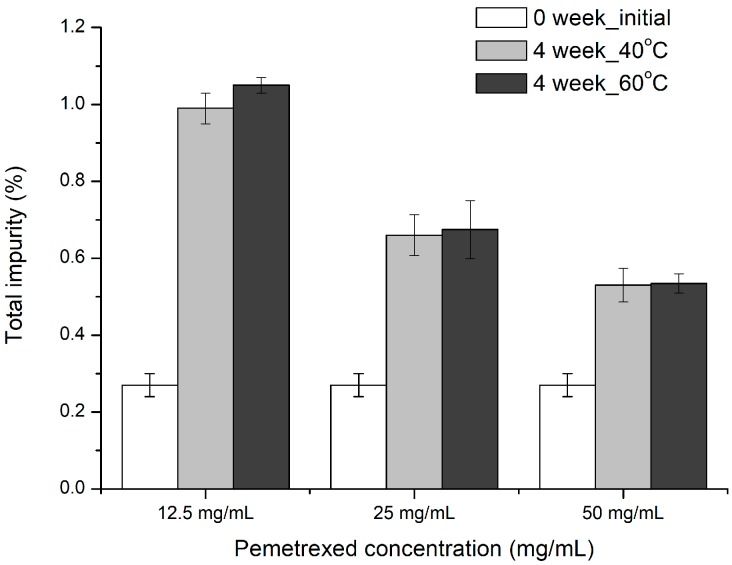
Effect of pemetrexed concentration on its degradation in aqueous solution induced by stress condition at 40 and 60 °C for four weeks.

As with all major degradation mechanisms, it was reported that the oxidation process was greatly affected by the drug concentration [21]. Therefore, the optimal drug concentration should be determined first, considering the process and cost efficiencies based on the stability. The total impurity (%) after the stability tests of pemetrexed solutions at various concentrations, as ranked by the SNK test, is as follows (*p* < 0.05): 12.5 mg/mL > 25 mg/mL > 50 mg/mL (Figure 5). This result shows a concentration-dependent degradation of pemetrexed in aqueous solution, which implies that higher concentrations are more favorable for stability. In general, the lower the concentration of the drug, the higher the probability of oxidative attack by dissolved oxygen. Consequently, considering the solubility and related probability of crystal precipitation, it was determined that 50 mg/mL was the optimal concentration for pemetrexed injectable solution.

#### 3.2.3. Effect of pH

**Figure 6:**
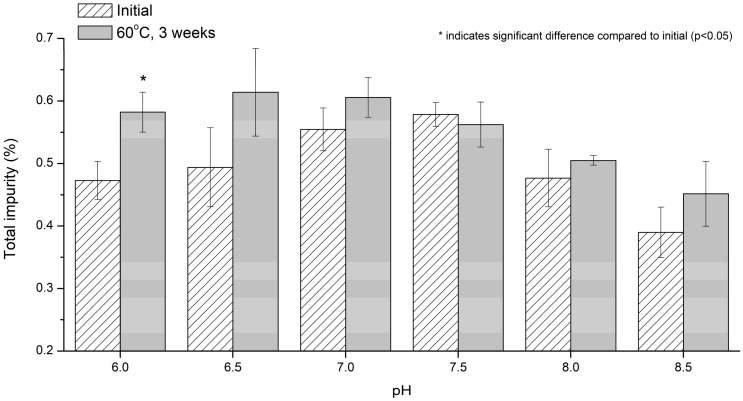
Effect of pH on the pemetrexed degradation induced by stress condition at 60 °C in aqueous solution. * indicate significant difference compared to inital (*p* < 0.05).

As shown in Figure 6, there was no significant difference observed in the total impurity (%) before and after pH stability testing over the pH range of 6.5–8.5. Despite the pH-dependent pemetrexed stability in solution, the reason for the stable state in the pH range of 6.5–8.5 was considered to be the addition of antioxidants. However, at pH 6, the total impurity level was significantly increased (*p* < 0.05), which implies that more degradation occurred than the ability to cover pemetrexed stability in the used formulation. The increased total impurity should be due to the des-glutamate and glutamic acid that were formed from the acid-catalyzed hydrolysis of the amide linkage of pemetrexed, and their further degradation by oxidation, as mentioned above [6,7].

### 3.3. Control of Process: Effect of Dissolved Oxygen (DO) Level

**Figure 7:**
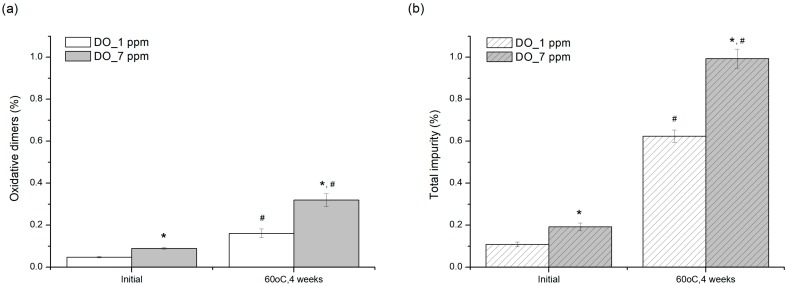
Effect of DO level (ppm) on pemetrexed degradation in aqueous solution induced by stress condition at 60 °C for four weeks: (**a**) increase in oxidative dimers and (**b**) increase in total impurity. * indicate significant difference compared to 1 ppm DO (*p* < 0.05); # indicate significant difference compared to initial value (*p* < 0.05).

Figure 7 shows that higher DO levels not only significantly induce the decomposition of pemetrexed in a short time from immediately after preparation of test sample to HPLC analysis, but also resulted in more pronounced degradation under stress conditions of 60 °C. The DO level in pemetrexed solution significantly influenced the oxidative reactivity of pemetrexed in this stability screening test; thus, it was confirmed that molecular oxygen in solution must be involved in the rate-limiting oxidation step [10]. From this result, it can be suggested that DO levels in pemetrexed injectable solution should be controlled at less than 1 ppm as low as possible.

### 3.4. Control of Packaging: Effect of Headspace Oxygen

**Figure 8:**
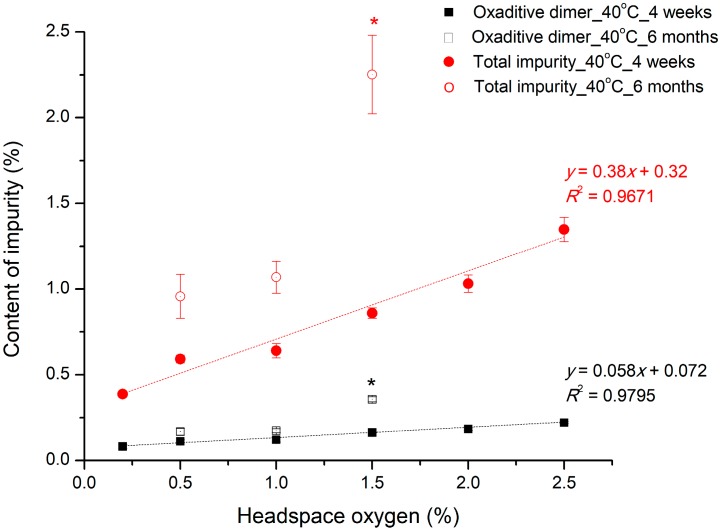
Effect of headspace oxygen level (%) on pemetrexed degradation in aqueous solution induced by accelerated condition at 40 °C for four weeks and six months. * indicate significant difference compared to 1.0% headspace oxygen sample (*p* < 0.05).

Whether the oxygen levels (%) in the headspace controlled by nitrogen headspace affect the stability of the pemetrexed in aqueous solution was investigated, and the results are shown in Figure 8. If the samples have been stored at 40 °C for four weeks, as the oxygen level in the headspace increases, the degradation products increase with a good linear correlation. This result indicates that the headspace environments have a significant effect on the oxidative reactivity of pemetrexed in aqueous solution. In addition, this result confirmed once again that molecular oxygen is involved in the rate-limiting oxidation step, similar to the effects of DO level, as discussed above [10]. It is known that an oxygen level between 3% and 8% in the headspace is generally attainable in most manufacturing sites. However, based on the results of this study, this range is considered too high and too wide to secure the stability of pemetrexed, suggesting that a tighter standard with narrow range of headspace oxygen level is necessary. To determine an optimal headspace oxygen level (%), the stability of the test samples with various headspace oxygen levels (%) was evaluated further at 40 °C for six months. The degradation product content increased dramatically from more than 1% of the headspace oxygen level, and there were no significant differences in the degradation between the samples below 1% of headspace oxygen level. In addition, it should be considered that the total impurity specification of pemetrexed for injection, as described in USP monograph, is less than 1.3%, even though it is a freeze-dried powder, not a solution. Hence, it was concluded that 1% of the headspace oxygen level was optimal to prevent oxidative degradation of pemetrexed efficiently in aqueous solution.

## 4. Conclusions

In this study, we investigated the effects of various parameters at each control strategy in drug product degradation on the stability of pemetrexed in injectable aqueous solution. A forced degradation study confirmed that oxidation is the main mechanism responsible for the degradation of pemetrexed in aqueous solutions. As formulation controls, the effects of the addition of antioxidants at various concentrations, drug concentration, and pH were evaluated; it was found that these parameters significantly influence the stability of the pemetrexed solution. In addition, the DO level in the solution controlled by nitrogen purging and oxygen level in headspace controlled by nitrogen headspace also showed significant effects on the stability of the pemetrexed solution; thus, it was confirmed that the molecular oxygen is involved in the rate limiting oxidation step. Based on the results obtained above on various control strategies, the optimal injectable solution formulation of pemetrexed is suggested as follows: sodium sulfite at 0.06 mg/mL, as an antioxidant for prevention of color change; NAC at 1.63 mg/mL, as an antioxidant for prevention of chemical degradation; pH range of 7–8; DO levels below 1 ppm; and headspace oxygen level below 1%. Therefore, it can be concluded that this study, which included well-designed control strategies, can lead to a better understanding of the complex degradation mechanism of pemetrexed; thus, it could contribute to the development of an injectable solution formulation of pemetrexed, with improved stability, which in turn may increase the utilization and safety of this drug. Furthermore, the results of this study can be used to set a standard criterion for degradation products of pemetrexed in injectable solution. In further studies, the optimal selection of container/closure with respect to minimizing the oxygen ingress based on the moisture vapor transmission rate (MVTR) should also be investigated as a key factor, considering a balance between the cost and protection efficiency.

## Figures and Tables

**Table 1:** HPLC elution program of the binary gradient system for pemetrexed analysis.

Time (min)	A ^1^ (%)	B ^2^ (%)
0	95	5
15	86	14
30	82	18
48	70	30
60	30	70
70	30	70
71	95	5
91	95	5

^1^ 1.36 g/L potassium dihydrogen phosphate with pH 2.5, ^2^ Acetonitrile.

**Table 2:** Compositions of stability test sample solutions.

Control Strategy	Factors	Antioxidants				Pemetrexed Conc. (mg/mL)	Mannitol Conc. (mg/mL)	pH	DO (ppm)	Headspace Oxygen (%)	Storage Condition
		Classification [29]		Name	Conc. (mg/mL)						
Formulation	Antioxidants	Oxidation reducing agents	Sulfide	Sodium sulfide	20	50	50	7	1	1	60 °C, 3 w
			Sulfite	Sodium sulfite	0.03, 0.06, 0.12	50	50	7	1	1	
				Sodium metabisulfite	0.06	50	50	7	1	1	
			Amino acids	NAC	1.00, 1.32, 1.63	50	50	7	1	1	
				l-cysteine·HCl	17	50	50	7	1	1	
		Phenolic	Vitamin	Vitamin E TPGS	0.7	50	50	7	1	1	
		Inclusion complex		HP-β-CD	20	50	50	7	1	1	
	Drug conc.	Oxidation reducing agents		Sodium sulfite	0.06	12.5, 25, 50	50	7	1	1	40 and 60 °C, 4 w
				NAC	1.63						
	pH	Oxidation reducing agents		Sodium sulfite	0.06	50	50	6–8.5	1	1	60 °C, 3 w
				NAC	1.63						
Process	DO ^1^	Oxidation reducing agents		Sodium sulfite	0.06	50	50	7	1, 7	1	60 °C, 4 w
				NAC	1.63						
Packaging	Headspace oxygen	Oxidation reducing agents		Sodium sulfite	0.06	50	50	7	1	0.2–2.5	40 °C, 4 w
				NAC	1.63						

^1^ Dissolved oxygen (DO) level in solution.

**Table 3:** HPLC elution program of the binary gradient system for NAC analysis.

Time (min)	A ^1^ (%)	B ^2^ (%)
0	100	0
7	100	0
8	50	50
13	50	50
14	100	0
28	100	0

^1^ 50 mM of KH_2_PO_4_ with pH 3.0, ^2^ Acetonitrile.

**Table 4:** Relative retention time (RRT) of pemetrexed’s degradation products.

Degradation Product	RRT		
	This Study	Literature [8]	USP
Pemetrexed	1	1	1
α-Hydroxy lactams	0.45	0.39/0.40	0.16/0.17
Ketopemetrexed	0.60/0.61	0.56/0.57	0.32
*N*-methyl derivative	0.74	0.79	0.76
Oxidative dimers	0.88	0.86/0.87	0.66
Gamma glutamate	0.90	0.88	0.70
Ring-opened keto-amide	0.93	0.91	0.80
Ring-opened keto-formamide	1.12	1.14	1.09
Epoxy hemiaminal	NA ^1^	0.23	0.08

^1^ Not applicable.
